# Outdoor activity and myopia progression in 4-year follow-up of Chinese primary school children: The Beijing Children Eye Study

**DOI:** 10.1371/journal.pone.0175921

**Published:** 2017-04-27

**Authors:** Yin Guo, Li Juan Liu, Ping Tang, Yan Yun Lv, Yi Feng, Liang Xu, Jost B. Jonas

**Affiliations:** 1 Beijing Institute of Ophthalmology, Beijing Key Laboratory of Ophthalmology and Visual Sciences, Beijing Tongren Eye Center, Beijing Tongren Hospital, Capital Medical University, Beijing, China; 2 Tongren Eye Care Center, Beijing Tongren Hospital, Capital Medical University, Beijing, China; 3 Department of Ophthalmology, Medical Faculty Mannheim of the Ruprecht-Karls-University Heidelberg, Mannheim, Germany; University of Houston, UNITED STATES

## Abstract

**Purpose:**

To investigate factors associated with ocular axial elongation and myopia progression during a 4-year follow-up in primary school children in Beijing.

**Methods:**

This school-based study included 382 grade-1 children at baseline in 2011 (age:6.3±0.4 years) with 305 (79.8%) returning for the follow-up examination in 2015. At baseline and in yearly follow-up examinations, the children underwent a comprehensive eye examination including auto-refractometry, ocular biometry with measurement of axial length, and fundus photography. The parents underwent a standardized interview.

**Results:**

During the study period, the mean axial length elongated by 1.15±0.56mm in boys and 1.10±0.63mm in girls. At baseline and at the end of follow-up, axial length was significantly (*P*<0.001) longer in boys, with no difference (*P* = 0.50) between genders in axial elongation. In multivariate analysis, greater axial elongation was associated (regression coefficient r^2^:0.15) with less time spent outdoors (*P* = 0.004; standardized coefficient beta: -0.22), more time spent indoors with studying (*P* = 0.02; beta: 0.18) and paternal myopia (*P* = 0.03; beta: 0.16). Larger increases in the axial length/anterior corneal curvature (AL/CC) ratio were associated (r^2^:0.09) with less time spent outdoors (*P*P = 0.003; beta: -0.22) and maternal myopia (*P*P = 0.02; beta: 0.18).

**Conclusions:**

Myopic axial elongation during a 4-year follow-up was associated with shorter time spent outdoors and longer time spent indoors studying and with parental myopia. Other factors such as level of paternal education, family income, gender and region of habitation were significantly associated with axial elongation and with myopia progression only in univariate analysis.

## Introduction

The prevalence of myopia has markedly increased in the past two decades in particular in East Asia. Recent studies have shown that approximately 80% to 90% of 18-year old school children are myopic, and 10% to 20% of the teenagers are highly myopic with a myopic refractive error of ≥-6 diopters [1–4]. There is also considerable evidence that similar trends are appearing in other parts of the world, including Europe and North America, where the prevalence of myopia has now reached 40–60% in younger adults [1,5]. Since high myopic refractive error is associated with pathologic myopia including myopic maculopathy and high myopia-associated glaucomatous optic neuropathy, intensive research has being conducted to search for factors associated with the marked myopic shift which took place within one generation [6–11]. These studies revealed that a higher prevalence of myopia among children and teenagers was significantly correlated with less time spent outdoors and the more time spent indoors, in addition to other factors such as female gender, higher prevalence and degree of parental myopia, higher parental level of education, and others [8–15].

Most previous studies were cross-sectional investigations, while only a minority of the studies already published assessed the progression of myopia and its associated factors in a longitudinal manner [16–22]. The population-based, longitudinal Sydney Adolescent Vascular and Eye Study included 2103 children, who underwent cycloplegic autorefractometry and who followed up for 5 to 6 years [19]. In that study, incident myopia was associated with less time spent outdoors and more near work carried out, after adjusting for parental myopia, East Asian versus European Caucasian ethnicity and a less hyperopic refractive error at study baseline. The association was present in particular in the younger group of study participants. The Avon Longitudinal Study of Parents and Children revealed that incident myopia, as measured by non-cycloplegic autorefractometry, was associated with both time spent outdoors and physical activity, with time spent outdoors having the larger effect [16]. In the Collaborative Longitudinal Evaluation of Ethnicity and Refractive Error Study however, time spent outdoors and outdoor sports activity were not associated with less progression of myopia [17]. In a similar manner, the amount of near work activity had little effect on the rate of myopia progression. In the study by Read and colleagues, 101 children with an age of 10 to 15 years underwent ocular biometry and were followed for a period of 18 months [22]. Less axial elongation was marginally significantly (*P* = 0.047) correlated with greater daily light exposure, in addition to more myopic refractive error, female gender and older age. In the study preceding the present investigation on the same study population, an increase in myopia during a 12-months follow-up was correlated associated with less time spent outdoors and more time spent indoors [20].

Since the preceding studies differed in their methodology, varying between non-cycloplegic refractometry and biometry, because the study period of the previous investigations was usually relatively short, and since the results were partially divergent, we carried out the present study to re-examine the change in axial length as anatomical surrogate for myopia in a longitudinal study design spanning a follow-up of 4 years.

## Methods

The Beijing Children Eye Study included primary students attending primary schools (grade 1) from a rural region and an urban area in Greater Beijing. The study protocol was approved by the Human Research Ethics Committee of the TongRen Hospital, Capital Medical University, Beijing. After explanation of the study design to parents and children, informed written consent was obtained from at least one parent per child. The study was conducted in 2011 and followed up once a year, which has been described in detail recently [15]. The two study sites were the urban Beijing Dong Cheng district with an average income of 30,684 Yuan in 2010 (average income across whole Beijing: 19640 Yuan) and located in the center of Beijing, and the rural Beijing Huai Rou district with an average income of 11,012 Yuan and located in the southeast of Beijing at a distance of 40 kilometers from the center of Beijing. While the baseline examination of the study in 2011 included pupils of grade 1 and grade 4, the present investigation included only the children who were in grade 1 in 2011, since the follow-up examination performed in 2015 was not sufficiently complete for the formerly grade 4 pupils.

All study participants underwent a comprehensive eye examination including measurement of visual acuity and auto-refractometry, assessment of ocular motility, slit lamp assisted biomicroscopy of the anterior and posterior segment of the eye, and non-mydriatic digital fundus photography (45°; CR-DGI camera, Canon Inc, Tokyo, Japan). Ocular biometric parameters (central corneal thickness, corneal curvature, anterior chamber depth, lens thickness, axial length) were measured for the right eye of all subjects by optical low-coherence reflectometry (Lenstar 900® Optical Biometer, Haag-Streit, 3098 Koeniz, Switzerland). The axial length / corneal curvature radius ratio (AL/CCR) was calculated. Refractometry was performed in a non-cycloplegic state by auto-refractometry (auto-refractor KR-8900, Topcon, Tokyo, Japan) followed by subjective refractometry. Since refractometry was carried out in non-cycloplegic condition, we used axial length and the AL/CCR ratio as additional surrogates for the refractive error. All examinations were undertaken by trained ophthalmologists and optometrists. The parents underwent an interview. The standardized questionnaire included inquiries about the time spent daily indoors or outdoors and which activities were performed at that time, and questions about the profession of the parents. We differentiated between professions characterized mostly by physical activities, such as farming or industry worker, and professions characterized mostly by less physical and more mental activities, such as clerk, teacher or manager. The same questionnaire, which had already been used and validated in the previous surveys performed in 2011 and each following year, consisted of questions on the children’s family history, time spent outdoors and the activities performed outdoors, time spent indoor and the activities carried out indoors, and studying for school. The average number of hours spent daily outdoors was calculated using the following formula: [(hours spent on a weekday)×5 +(hours spent on a weekend day)×2]/7 [15]. The total outdoor activity was defined as the sum of outdoor leisure and outdoor sports. We additionally asked about the birth weight, being breast fed, smoking and alcohol consumption of father and mother, level of education and profession of the parents, size of the house (in m^2^), monthly family income, and prevalence of myopia of the parents. The same examinations were performed at the baseline in 2011 and they were repeated in the follow-up examination in 2015.

The size of the optic disc and optic cup was measured on the optic disc photographs, using Image J software (version 1.43u; developed by Wayne Rasband, National Institutes of Health, Bethesda, MD; available in the public domain at http://rsb.info.nih.gov/ij/index.html). The parapapillary regions alpha and beta were assessed as described previously [23,24]. Alpha zone was characterized by an irregular hypopigmentation and hyperpigmentation, and intimated thinning of the chorioretinal tissue layer. On its outer side it was adjacent to the retina, and on its inner side it was in touch with beta zone, or if the beta zone was not present, with the peripapillary ring of the optic disc margin. Features of beta zone were a good visibility of the large choroidal vessels and / or of the sclera, thinning of the chorioretinal tissues and round bounds to the adjacent alpha zone on its peripheral side and to the peripapillary ring on its central side. The magnification by the optic media of the eye was corrected by applying the method of Littmann, using either the axial length measurements or the refractive error measurements [25]. The measurements were carried out by a trained ophthalmologist (YG) supervised by a panel of glaucoma specialists (LX, JBJ).

Additionally, we measured body height and weight and calculated the body mass index (BMI) using the formula of BMI = Body Weight / ((Body Height in m) + (Body Height in m)). The body height was measured with the shoes routinely removed. The children were asked to stand upright as much as possible and with the head raised upright as much as possible. We used a stadiometer as measuring instrument.

Statistical analysis was performed using a commercially available statistical software package (SPSS for Windows, version 22.0, SPSS, Chicago, IL). The data of the right eyes was analyzed. Myopia was defined as a refractive error (spherical equivalent) of ≤-1.00 diopter. Children with incident myopia were not myopic at baseline and had become myopic at the end of follow-up. The cumulative incidence of myopia was defined as the proportion of children who were not myopic at baseline and who subsequently developed myopia during the follow-up period. To examine potential associations between environmental factors and the development or progression of myopia, we first calculated changes in axial length, refractive error and the AL/CC ratio as difference between the measurements obtained at baseline in 2011 and the readings taken at the last follow-up examination in 2015. For this calculation, we did not consider the measurements obtained in between unless the readings taken in 2015 did not fit in line with the measurements taken previously. If the value of axial length measurement performed in 2015 was shorter than the one of measurement carried out at baseline in 2011, we compared the measurements obtained in 2012, 2013 and 2014. If all these values except for the last value measured in 2015 indicated an axial elongation, we took the value measured in 2014 for the statistical analysis. This procedure was performed for about 3 (1%) study participants. The changes in these main outcome parameters together with the incidence rate of myopia were then correlated with the baseline measures of the other parameters such as the results of the questionnaire and the optic disc measurements. The parameters were presented as mean ± standard deviation. The normal distribution of parameters was examined using the Kolmogorov-Smirnoff test. After univariate analysis of potential associations, we performed a stepwise multivariate regression analysis with the myopia-related oculometric parameters (i.e. axial elongation and increase in the AL/CC ratio) as dependent variables, and all parameters as independent variables which showed a significant association with the main outcome parameter in the univariate analysis. Standardized regression coefficient beta and the non-standardized regression coefficient B and its 95% confidence intervals (CI) were calculated. The associations with incidence of myopia were examined in a binary regression analysis and odds ratios (OR) were calculated. All *P*-values were 2-sided and were considered statistically significant when the values were less than 0.05.

## Results

At baseline in the year 2011, the study included 382 grade-1 students with a mean age of 6.3 ± 0.4 years (range: 5–8) and a mean refractive error of the right eyes of -0.23 ± 1.01 (median: -0.13 diopters; range: -6.75 to +4.88 diopters). Out of these 382 children, 216 (56.5%) students were living in the urban region. Mean axial length measurements available for 368 (96.3%) children was 23.2 ± 0.9 mm in the boys and 22.8 ± 1.0 mm in the girls with a significant difference (*P*<0.001) between the sexes.

Out the 382 students, 305 (79.8%) children returned for the follow-up examination in 2015. Best corrected visual acuity, measured in the negative decadic logarithm of the angle of spatial resolution (logMAR), was 0.00 ± 0.04 logMAR (median: 0.00; range: 0.00 to 0.40) for the right eyes and 0.01 ± 0.04 logMAR (median: 0.00; range: 0.00 to 0.40) for the left eyes. Presenting visual acuity was 0.07 ± 0.11 logMAR (median: 0.00; range: 0.00 to 0.40) for the right eyes and 0.06 ± 0.09 logMAR (median: 0.00; range: 0.00 to 0.40) for the left eyes. Mean refractive error had changed to -1.21 ± 1.76 diopters (median -0.88 diopters; range: -7.00 to +4.50 diopters) for right eyes and to -1.10 ± 1.83 diopters (median: -0.63 diopters; range: -7.50 to +6.50 diopters) in the left eyes. Axial length in the right eyes measured 23.8 ± 1.1 mm (median: 23.8 mm; range: 21.1–27.0 mm) with a significant (*P*<0.001) difference between boys (24.1 ± 1.1 mm) and girls (23.5 ± 1.0 mm). The prevalence of myopia was 17.3% (66/382) (95% CI: 13.5, 21.2). Mean time spent daily outdoors with sports was 0.7 ± 0.1 hours and spent outdoors with leisure was 1.0 ± 0.8 hours, totaling 1.7 ± 0.8 hours daily. The mean time spent daily indoors with studying was 5.2 ± 0.8 hours, the mean time spent with viewing television was 0.1 ± 0.2 hours, and the mean time spent daily with electronic gadgets was 0.8 ± 0.8 hours. The time spent for studying indoors was reciprocally correlated with the time spent outdoors (*P*<0.001; beta: -0.30).

### Axial elongation

During the study period, the mean axial length increased by 1.15 ± 0.56 mm to 24.1 ± 1.1 mm in the boys and by 1.10 ± 0.63 mm to 23.5 ± 1.0 mm in the girls. At the end of the follow-up, axial length remained to be significantly (*P*<0.001) longer in the boys than in the girls. Both sexes did not differ significantly (*P* = 0.50) in the amount axial elongation. The axial elongation differed significantly (*P*<0.001) between the rural region (0.84 ± 0.56 mm) and the urban region (1.32 ± 0.54mm).

**Fig 1:**
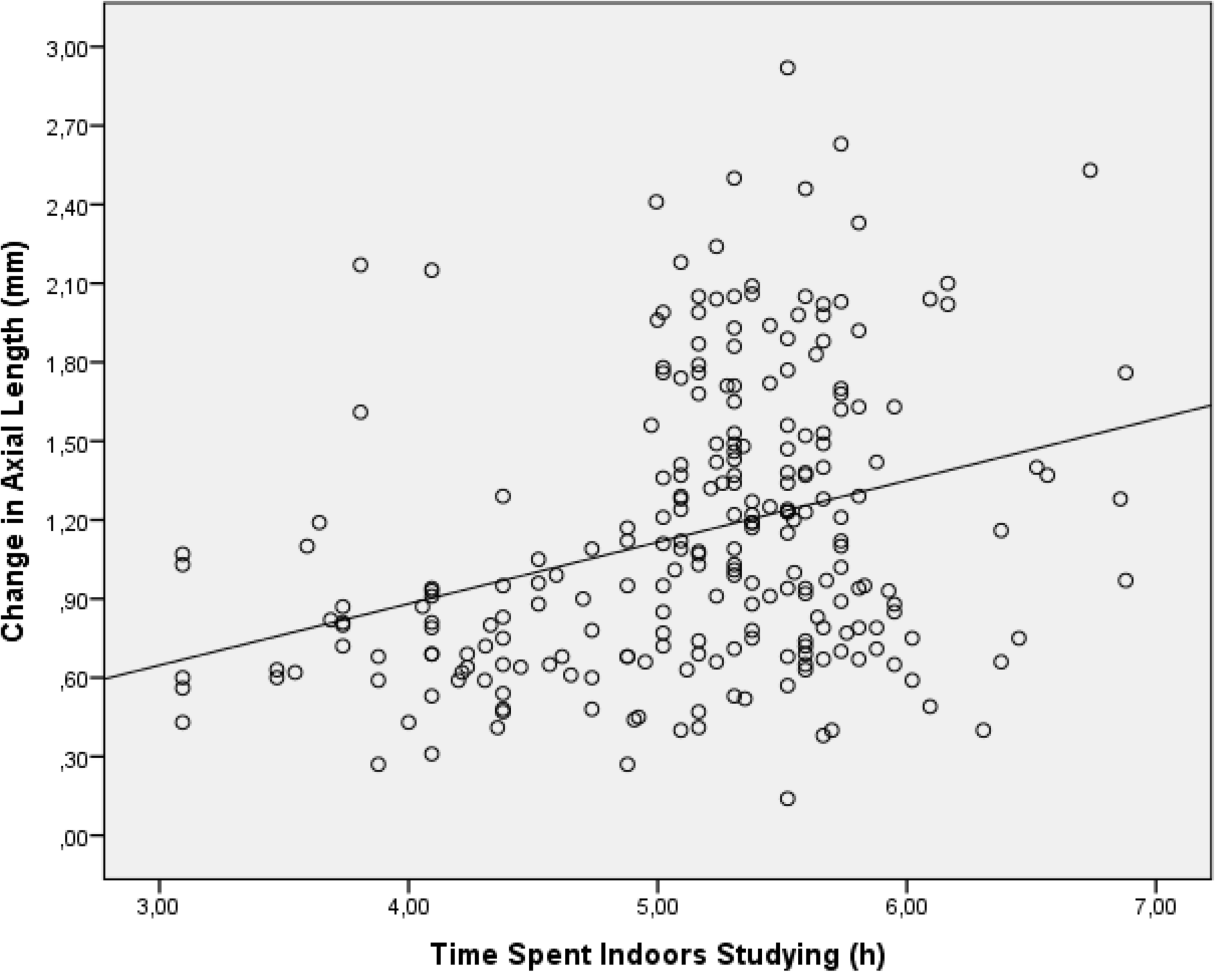
Graph showing the distribution of the change in axial length versus time spent indoors studying.

In univariate analysis, the axial elongation was significantly associated with urban region of habitation, higher level of education of father and mother, mental versus physical occupation of father and mother, higher family income, myopia of father and mother, smoking of the father, less outdoors time spent with leisure and less total time spent outdoors, more indoor time spent with studying (Fig 1), more time watching television, axial length, anterior chamber depth, lens thickness and refractive error at baseline, and the area of the optic disc and optic cup at baseline. The amount of axial elongation was not significantly associated with age, gender, higher body height and weight, BMI, birth weight, history of breast feeding, self-reported smoking of the father and mother during pregnancy, alcohol consumption by the father or mother, horizontal optic disc diameter, parapapillary beta zone area and parapapillary alpha zone area (Table 1).

**Table 1:** Associations (univariate analysis) between axial elongation and ocular and systemic parameters in The Beijing Children Eye Study.

Parameter	*P*-Value	Standardized Regression Coefficient Beta	Non-Standardized Regression Coefficient B	95% Confidence Interval
Age (Years)	0.76			
Gender	0.49			
Maternal Myopia	0.01	0.15	0.22	0.05, 0.38
Paternal Myopia	0.003	0.18	0.23	0.08, 0.38
Birth Weight (kg)	0.16			
Mother´s Occupation (Blue Collar / White Collar)	<0.001	0.27	0.31	0.16, 0.45
Father´s Occupation (Blue Collar / White Collar)	0.000	0.30	0.36	0.22, 0.50
Smoking by Mother	0.31			
Smoking by Father	0.33			
Education of Mother	<0.001	0.22	0.17	0.08, 0.26
Education of Father	<0.001	0.26	0.24	0.13, 0.34
Family income	<0.001	0.28	0.11	0.07, 0.16
Body Height (cm)	0.29			
Body Weight (kg)	0.76			
Body Mass Index (kg/m^2^)	0.69			
Urban / Rural Region of Habitation	<0.001	-0.41	-0.49	-0.61, -0.36
Time Spent Outdoors (Hours) in 2011	<0.001	-0.28	-0.22	-0.34, -0.11
Time Spent Outdoors Leisure (Hours) in 2011	0.001	-0.29	-0.23	-0.34, -0.12
Time Spent Outdoors Sports (Hours) in 2011	0.04	-0.14	-0.77	-1.48, -0.05
Time Spent Indoors Studying (Hours) in 2011	<0.001	0.29	0.21	0.12, 0.30
Time Spent Indoors Watching (Hours) in 2011	<0.001	-0.27	-0.20	-0.29, -0.11
Parapapillary Beta Zone Area (mm^2^)	0.84			
Parapapillary Alpha Zone Area (mm^2^)	0.59			
Axial Length (mm)	0.007	0.15	0.11	0.03, 0.20
Refractive Error (Diopters)	<0.001	-0.29	-0.16	-0.22, -0.10

The multivariate analysis included axial elongation as the dependent variable and all parameters as independent variables which were significantly associated with axial elongation in the univariate analysis. Due to collinearity, we first dropped time spent outdoors with leisure (variance inflation factor (VIF): 17.6), region of habitation (VIF: 4.4), paternal level of education (variance inflation factor (VIF): 3.9) and maternal occupation (VIF: 3.3) from the list of independent variables. Due to a lack of statistical significance, we then dropped maternal level of education (*P* = 0.83), income (*P* = 0.80), smoking of father (*P* = 0.31), axial length (*P* = 0.99), lens thickness (*P* = 0.77), time watching television (*P* = 0.85), optic disc area (*P* = 0.74), paternal occupation (*P* = 0.43), anterior chamber depth (*P* = 0.35), refractive error (*P* = 0.21), area of the optic cup (*P* = 0.07), and maternal myopia (*P* = 0.08). In the final model, a larger amount of axial elongation during the study period was associated (regression coefficient r^2^: 0.15) with less time spent outdoors overall (*P*P = 0.003), more time spent indoors with studying (*P*P = 0.03) and with paternal myopia (*P*P = 0.02) (Table 2). If the parameters of time spent indoors and time spent outdoors were replaced by the parameter of the ratio of total time spent indoors / total time spent outdoors or by the parameter of the ratio of time spent indoors studying / total time spent outdoors, the regression coefficient (r^2^: 0.11 and r^2^: 0.15, respectively) did not improve.

**Table 2:** Associations (multivariate analysis) between the axial elongation and ocular and systemic parameters in The Beijing Children Eye Study.

Parameter	*P*-Value	Standardized Regression Coefficient	Non-Standardized Regression Coefficient	95% Confidence Interval
Time Spent Outdoors (Hours)	0.004	-0.22	-0.09	-0.15, -0.03
Time Spent Indoors Studying (Hours)	0.03	0.16	0.12	0.01, 0.22
Paternal Myopia	0.02	0.17	0.24	0.03, 0.44

### Increase in axial length/ corneal curvature radius (AL/CC)

The mean AL/CC ratio was increased by 0.14 ± 0.07 from 2.94 ± 0.1 to 3.04 ± 0.12. The increase in the AL/CC ratio was significantly (*P*<0.001) more marked in the urban region (0.17 ± 0.07) than in the rural region (0.10 ± 0.06).

In univariate analysis, the increase in AL/CC was significantly associated with urban versus rural region of habitation, higher level of education of father and mother, mental versus physical occupation of father and mother, higher family income, myopia of father and mother, smoking by mother, less total time spent outdoors, less outdoors time spent with leisure, more indoor time spent with studying, more time watching television. Change in the AL/CC ratio was not significantly associated with age, gender, body height and weight, birth weight, history of breast feeding, self-reported smoking of the father and mother, alcohol consumption by father and mother, time spent outdoors with sports and time spent with playing with electronic gadgets (Table 3).

**Table 3:** Associations (univariate analysis) between the increase in the ratio of axial length / corneal curvature radius and ocular and systemic parameters in The Beijing Children Eye Study.

Parameter	*P*-Value	Standardized Regression Coefficient	Regression Coefficient	95% Confidence Interval
Age (Years)	0.69			
Gender	0.95			
Maternal Myopia	<0.001	0.21	0.034	0.015, 0.054
Paternal Myopia	0.008	0.16	0.029	0.007, 0.050
Birth Weight (kg)	0.30			
Mother´s Occupation (Blue Collar / White Collar)	<0.001	0.28	0.044	0.024, 0.064
Father´s Occupation (Blue Collar / White Collar)	<0.001	0.29	0.045	0.027, 0.063
Smoking by Mother	0.045	-0.13	-0.044	-0.086, -0.001
Smoking by Father	0.08			
Education of Mother	<0.001	0.25	0.025	0.014, 0.037
Education of Father	<0.000	0.30	0.034	0.021, 0.048
Family income	<0.001	0.32	0.017	0.011, 0.023
Body Height (cm)	0.29			
Body Weight (kg)	0.98			
Body Mass Index (kg/m^2^)	0.83			
Urban / Rural Region of Habitation	<0.001	-0.42	-0.064	-0.080, -0.048
Time Spent Outdoors	0.001	-0.25	-0.024	-0.038, -0.011
Time Spent Outdoors Leisure	<0.001	-0.26	-0.025	-0.038, -0.012
Time Spent Outdoors Sports	0.06			
Time Spent Indoors Studying	<0.001	0.26	0.024	0.012, 0.035
Time Spent Watching Television	<0.001	-0.24	-0.023	-0.034, -0.011
Beta PPA Area (mm2)	0.71			
Alpha PPA Area (mm2)	0.30			

Due to collinearity in the multivariate analysis with change in AL/CC ratio as dependent parameter, we first dropped paternal level of education (variance inflation factor (VIF): 3.4) and region of habitation (VIF: 3.3) from the list of independent variables. Due to a lack of statistical significance, we then dropped maternal occupation (*P* = 0.90), paternal occupation (*P* = 0.91), smoking by the mother (*P* = 0.47), maternal level of education (*P* = 0.93), time watching television (*P* = 0.23), time spent outdoors with sports (*P* = 0.14), income (*P* = 0.09), paternal myopia (*P* = 0.06), and time spent indoors with studying (*P* = 0.08). In the final model, a larger increase in the AL/CC ratio during the study period was associated (r^2^: 0.09) with less time spent outdoors (*P*P = 0.003; beta: -0.22; B: -0.02; 95%CI: -0.04, -0.01) and with maternal myopia (*P*P = 0.02; beta: 0.18; B: 0.03; 95%CI: 0.01, 0.05).

### Incidence of myopia

The accumulative incidence of myopia was 35.1% (134/382) during the study period of 4 years. The 4-year change in spherical equivalent refraction was −0.92 ± 1.51 diopters.

In univariate analysis, the incidence of myopia was significantly associated with urban versus rural region of habitation, higher level of education of father and mother, mental versus physical occupation of father, myopia of father and mother, mental versus physical occupation of mother, higher family income, maternal age of mother, less total outdoors time, less outdoors time spent with leisure, more indoors time spent with studying, indoors time spent with watching television. Incidence of myopia was not significantly associated with age, gender, history of breast feeding, self-reported smoking of the mother during pregnancy, self-reported alcohol consumption by father and mother, birth weight, outdoors time spent on sports, indoors time spent with watching television, time spent playing with electronic gadgets, beta PPA area and alpha PPA area (Table 4).

**Table 4:** Associations (univariate analysis) between incidence of myopia and ocular and systemic parameters in The Beijing Children Eye Study.

Parameter	*P*-Value	Odds Ratio	Regression Coefficient	95% Confidence Interval
Age (Years)	0.45			
Gender	0.77			
Maternal Myopia	0.02	1.96	0.67	1.14, 3.37
Paternal Myopia	0.01	2.19	0.78	1.20, 3.97
Birth Weight (kg)	0.49			
Maternal Age at Birth (Years)	0.005	1.09	0.08	1.03, 1.15
Mother´s Occupation (Blue Collar / White Collar)	0.002	2.47	0.90	1.41, 4.31
Father´s Occupation (Blue Collar / White Collar)	0.003	2.13	0.75	1.29, 3.49
Smoking by Mother	0.10			
Smoking by Father	0.21			
Education of Mother	0.005	1.61	0.48	1.16, 2.25
Education of Father	0.005	1.72	0.54	1.18, 2.50
Family income	0.002	1.30	0.26	1.10, 1.53
Body Height (cm)	0.39			
Body Weight (kg)	0.57			
Body Mass Index (kg/m^2^)	0.56			
Urban / Rural Region of Habitation	<0.001	0.29	-1.24	0.18, 0.46
Time Spent Outdoors	0.03	0.62	-0.47	0.41, 0.95
Time Spent Outdoors Leisure	0.005	0.56	-0.59	0.37, 0.84
Time Spent Outdoors Sports	0.87			
Time Spent Indoors Studying	0.04	1.38	0.320	1.02, 1.86
Time Spent Watching	0.004	0.61	-0.49	0.44, 0.86
Beta PPA Area (mm2)	0.07			
Alpha PPA Area (mm2)	0.75			

In the multivariate analysis, only the parameter of less time spent outdoors (OR: 0.630; 95%CI: 0.41, 0.98; *P* = 0.038) was significantly associated with a higher incidence of myopia.

## Discussion

Our school-based longitudinal observational study revealed that greater axial elongation during a follow-up of 4 years in Chinese primary school children was associated predominantly with shorter time spent outdoors and longer time spent indoors studying as well as with parental myopia. Other factors such as level of education of mother and father, family income, gender and region of habitation were significantly associated with axial elongation and with the progression of myopia only in univariate analysis.

These results confirm findings obtained in previous cross-sectional studies and a prospective trial [1–4,8–21]. The mean increase in axial length of 1.13 ± 0.58 mm and in the ALCC ratio of 0.14 ± 0.08 in our study population within four years was similar to the increase in axial length observed by Fan and colleagues in 307 preschool children with an age of 2–6 years, who showed a mean axial elongation of 1.72 mm within a 5 year period [26]. Similar measurements were made by Donovan and colleagues and Fujiwara and associates [27,28].

The findings of our study on the association between shorter time spent outdoors and longer time spent indoors agree with the observations made in other, mostly cross-sectional, investigations on children and teenagers [8–22]. In 2002, Mutti and associates reported on associations between myopia in children and parental myopia, higher amount of time spent for studying and reading, smaller amount of time spent with playing sports, and higher scores in school achievements [8]. Jones and colleagues from the same group of authors later examined survey-based data of 514 children from the Orinda Longitudinal Study of Myopia which was conducted between 1989 to 2001 [29]. They found that, besides parental myopia being an important risk factor for becoming myopic, less time spent for sports and for outdoor activity increased the risk of developing myopia more markedly in children with two myopic parents than in children with at maximum one parent being myopic. The probability of developing myopia for children with no myopic parents was lowest in children with the highest amount of time spent for sports and other outdoor activity. In the Collaborative Longitudinal Evaluation of Ethnicity and Refractive Error (CLEERE) Study, Jones-Jordan and associates examined 835 myopes and reported that neither the amount of time spent for reading for pleasure, the amount of time spent for other near activities or the time spent for outdoors for or sport activities were correlated with the progression of myopia with a level of significance of *P*<0.01 [17]. In the multivariate analysis, the largest effect found was that each additional 10 hours of reading for pleasure per week was associated with an increase in myopic refractive error of -0.08 diopters. It has remained unclear why in Jones-Jordan´s study the amount of time spent outdoors did not have a significant effect on the progression of myopia. Reasons for the differences in the results of various studies may have been differences in the type of questions and differences in the general behavior and life style of the population in the study region (e.g., rural population versus urban or metropolitan population). Guggenheim and colleagues reported that both, shorter time spent outdoors and less physical outdoors activity were associated with incident myopia, with time spent outdoors having the larger effect [30]. The authors concluded that the amount of time spent outdoors was the main influential factor for the incidence of myopia independently of the level of physical activity. The finding of an association between outdoors activity and myopia was also reported for other ethnic groups, such as from Jordan and from Turkey [31,32]. In these and other investigations, an increasing prevalence of myopia and of high myopia was associated with less time spent outdoors and more time spent indoors, with higher type of schools attended, higher prevalence of parental myopia, higher level of education and higher socioeconomic standing of the parents, and urban versus rural region of habitation. These investigations include the recent landmark study by He and colleagues in which school children spending more time outdoors as compared to school children spending less time outdoors developed significantly less often myopia as measured by refractometry [21]. In a study performed by Read and colleagues, lower amount of axial elongation was marginally significantly (*P* = 0.047) correlated with greater daily light exposure, in addition to more myopic refractive error, female gender and older age [22]. Read´s study was the first longitudinal and objective study of the potential association between light exposure and axial elongation. The authors discussed that the greater daily light exposure might have inhibited axial elongation, supporting the notion of light exposure, in addition to the amount of time spent outdoors and myopia at baseline, playing a role in the process of myopic axial elongation. It may have remained unclear however, whether the higher amount of light exposure was a surrogate of the time spent outdoors.

The reasons for the association between increasing axial elongation and less time spent outdoors and more time spent indoors have remained elusive yet. One has discussed the influence of greater light exposure (as also discussed by Read and colleagues), greater exposure to ultraviolet light, and less accommodation on near objects when staying outdoors as compared to studying indoors [8–22,33] The correlation between parental myopia and axial elongation in the children may be due to a hereditary component or the myopia of the parents might have been a surrogate of more studying of the children, since myopia in adults in China is associated with a higher level of education and higher family income [34].

The previous studies suggested that besides a higher amount of time spent indoors, other factors might additionally play a role in the association with myopia. In multivariate analyses, a higher prevalence of myopia in the children and teenagers was associated with urban versus region of habitation, ethnic background, higher parental myopia, higher level of education of the parents, more near work performed by the children, and better school achievements and higher level of schools attended by the children and teenagers [2,8–22]. It is parallel to our study in which most of these factors were associated with an increased axial elongation, however only in univariate analysis. Other studies doubted an association between myopia and ethnic background or the association between myopia in children and near work [14,35]. Our longitudinal study covering a relatively long follow-up period of 4 years showed that axial elongation, which has been reported to be show a strong association with the development of myopic retinopathy, was mainly dependent on the time spent outdoors and indoors and with parental myopia, while other factors such as level of paternal education, family income, gender and region of habitation were significantly associated with axial elongation and with myopia progression only in univariate analysis [36]. It may suggest that the major factors influencing the axial elongation in children and teenagers are the amount of time spent indoors and the amount of time spent outdoors as well as parental myopia. If this notion is valid, the level of education and the intensity of studying as compared to staying predominantly indoors or outdoors may be less strongly associated with the development and progression of myopia. It suggests that a compromise in the level of education and intensity of studying to prevent the development of high myopia may not be necessary.

Potential limitations of our study should be mentioned. First, the study was not population-based so that the possibility of a selection bias existed. Second, refractometry was not performed under cycloplegic conditions, so that involuntary accommodation during refractometry may have covered latent hyperopia. The main outcome parameter of our study was, however, axial length the measurement of which is independent of the accommodative status of the lens. Third, limitations associated with the interview of the parents and the questionnaire come into play since one of the main outcome parameters, time spent outdoors and indoors, was assessed by interviewing the children. It has to be taken into account that not all parents are well informed about the activities of their children, in particular during the period of puberty. Direct measurement of the time spent outdoors would have been a more objective and reliable method to get the information about the lifestyle of the children. Fourth, the habitual refractive correction worn by the children was not assessed so that it leaves open the possibility that an undercorrection of the refractive error could potentially have confounded the axial elongation data.

In conclusion, our longitudinal study spanning a follow-up period of 4 years is in agreement with previous cross-sectional investigations and with trials that less time spent outdoors and more time spent indoors is associated with a more marked axial elongation and progression of myopia in children and teenagers.
